# Vitamin B6, vitamin B12 and methionine and risk of pancreatic cancer: a meta-analysis

**DOI:** 10.1186/s12937-020-00628-7

**Published:** 2020-10-04

**Authors:** Dan-Hong Wei, Qi-Qi Mao

**Affiliations:** 1 Department of Neuroscience Care Unit, The Second Affiliated hospital, School of Medicine, Zhejiang University, Hangzhou, 310009 China; 2 Department of Urology, The First Affiliated hospital, School of Medicine, Zhejiang University, Hangzhou, 310003 China

**Keywords:** Meta-analysis, Methionine, Pancreatic cancer, Vitamin B6, Vitamin B12

## Abstract

**Background:**

Nutrients involved in one-carbon metabolism may play a key role in pancreatic carcinogenesis. The aim of this study was to examine the association between pancreatic cancer risk and intake or blood levels of vitamins B6, B12 and methionine via meta-analysis.

**Methods:**

A systematic search was performed in PubMed, Web of Knowledge and Chinese National Knowledge Infrastructure (CNKI) up to April 2020 to identify relevant studies. Risk estimates and their 95% confidence intervals (CIs) were retrieved from the studies and combined by a random-effect model.

**Results:**

A total of 18 studies were included in this meta-analysis on the association of vitamin B6, B12 and methionine with pancreatic cancer risk. The combined risk estimate (95% CI) of pancreatic cancer for the highest vs lowest category of vitamin B6 intake and blood pyridoxal 5′-phosphate (PLP, active form of vitamin B6) levels was 0.63 (0.48–0.79) and 0.65 (0.52–0.79), respectively. The results indicated a non-linear dose-response relationship between vitamin B6 intake and pancreatic risk. Linear dose–response relationship was found, and the risk of pancreatic cancer decreased by 9% for every 10 nmol/L increment in blood PLP levels. No significant association were found between pancreatic cancer risk and vitamin B12 intake, blood vitamin B12 levels, methionine intake and blood methionine levels.

**Conclusion:**

Our study suggests that high intake of vitamin B6 and high concentration of blood PLP levels may be protective against the development of pancreatic cancer. Further research are warranted to confirm the results.

## Introduction

Pancreatic cancer is one of the most lethal cancer worldwide, with an estimated 458,918 new cases and 432,242 deaths in 2018 [1]. Despite advances in treatment, the prognosis of pancreatic cancer is very poor, with a 5-year survival rates of 4% [2]. Because of poor prognosis and lack of effective screening methods for early detection, primary prevention is the only approach to reduce the burden of pancreatic cancer [3]. Smoking and obesity are established risk factors [4, 5]. Diet have been postulated to play a significant role in the development of pancreatic cancer and several biological mechanisms may explain the relationship between diet and pancreatic cancer risk [6].

Potential risk factors for pancreatic cancer are dietary nutrients associated with one-carbon metabolism, such as folate, vitamin B6, vitamin B12 and methionine, which may protect against cancer through DNA methylation, nucleotide synthesis, DNA replication and repair. For example, folate may influence gene stability and expression through its essential role in methionine synthesis and in the conversion to S-adenosylmethionine (SAM, the universal donor for DNA methylation), and vitamin B12 serves as a cofactor in this biochemical reaction [7, 8]. Vitamin B6 is a cofactor for multiple critical enzymes in the methyl-group metabolism pathway [9]. Lack of folate and other methyl-group nutrients may increase the risk of pancreatic cancer by altering the methylation of DNA and RNA, disrupting DNA integrity and DNA repair, increasing DNA damage and gene mutations [10, 11].

Many epidemiologic studies have assessed one-carbon metabolism-related nutrients associated with pancreatic cancer risk. A significant protective effect of folate on pancreatic cancer was reported in the previous meta-analysis [12, 13]. Due to the involvement of multiple nutrients and the complexity of one-carbon metabolism pathways, a comprehensive assessment of the nutrients involved and their relationship with risk of pancreatic cancer is needed. However, results are not inconsistent in the studies that have examined the association between vitamin B6, vitamin B12 and methionine and pancreatic cancer risk [14, 15], and no meta-analysis is available. In addition, several studies also analyzed blood levels of vitamin B6, vitamin B12 and methionine in relation to risk of pancreatic cancer [16, 17]. A blood biomarker approach would provide insights on the potential role of the intake of vitamins in the development of pancreatic cancer. In this study, we evaluate the evidence from observational studies on vitamin B6, B12 and methionine and the risk of pancreatic cancer by summarizing it quantitatively with a meta-analytic approach.

## Methods

### Literature search and selection

This meta-analysis follows the standards of quality for reporting systematic review and meta-analysis (PRISMA) [18]. A literature search up to April 2020 was performed using the PubMed, Web of Knowledge and Chinese National Knowledge Infrastructure (CNKI) with the following search terms in the free text: vitamin B6 or pyridoxal 5′-phosphate (PLP, the active form of vitamin B6) or vitamin B12 or methionine, and pancreatic cancer in the full text with no language limitation. The potentially relevant studies were assessed by screening their titles and abstracts. Full texts for articles matching the eligible criteria were retrieved. Moreover, the references from retrieved articles were hand searched for further relevant studies.

Two reviewers independently reviewed all identified studies, and studies were included in this meta-analysis if they met all the following criteria: (1) case-control, nested case-control study, cohort study design, or randomized controlled trials (RCTs); (2) the exposure of interest was intake of vitamin B6, vitamin B12 or methionine, or serum or plasma levels of them; (3) reported risk estimate and its 95% confidence interval (CI). The exclusion criteria were as follows: 1) experimental study; 2) letters or case reports; 3) articles that provided inadequate data or only information for cancer mortality. If multiple studies from the same general population were available, the study with the largest number of cases was included in this meta-analysis.

Data were extracted from each study by two reviewers. For each study, the following information were collected: first author’s name, publication year, the country in which the study was conducted, study design, number of cases, sex, doses, adjusted variables, type of exposure (dietary intake or blood level), and adjusted risk estimates for highest versus lowest level of vitamin B6 or vitamin B12 or methionine, or blood level of them. The result for dietary intake was extracted if both dietary intake and total intake (dietary intake plus supplement) were provided. Considering that pancreatic cancer is a rare disease, the relative risk (RR) was assumed approximately the same as OR, and the RR was used as the study outcome.

The quality of each study was assessed using the Newcastle-Ottawa Scale (NOS), which was recommended by the Cochrane Non-Randomized Studies Methods Working Group (http://www.ohri.ca/programs/clinical_epidemiology/oxford.asp). NOS is an eight-item instrument that is used for assessment of the study population, study comparability, follow-up and outcome of interest. The range of possible scores is 0–9, and we assigned scores of < 7 and ≥ 7 for low- and high-quality studies, respectively.

Random-effects model was used to compute a combined RR with its 95% CI, which considers both within and between-study variation. Heterogeneity between studies was assessed by Q statistic and the I^2^ score, and I^2^ values of 0, 25, 50 and 75% represent no, low, moderate and high heterogeneity, respectively [19]. A sensitivity analysis was performed with one study removed at a time to assess whether the results could have been affected markedly by a single study. Publication bias was evaluated by both Begg’s test [20] and Egger’s test [21]. For the dose-response analysis, we used a previously described method described by Greenland [22] and Orsini [23]. For studies that reported the intake by ranges of intake we estimated the midpoint in each category by calculating the average of the lower and upper bound. When the highest category was open ended, we assumed the length of the open-ended interval to be the same as that of the adjacent interval. When the lowest category was open-ended, we set the lower boundary to zero. A potential non-linear dose-response relationship was also explored by using restricted cubic regression splines with three knots at the 25th, 50th, and 75th percentiles of the distribution. A likelihood ratio test was used to assess the difference between the non-linear and linear models to test for non-linearity. All reported probabilities (*P*-values) were two-sided with *P* < 0.05 considered statistically significant. All the above statistical analyses were carried out with STATA 12.0 (StataCorp, College Station, TX, USA).

## Results

### Literature search and study characteristics

**Fig. 1:**
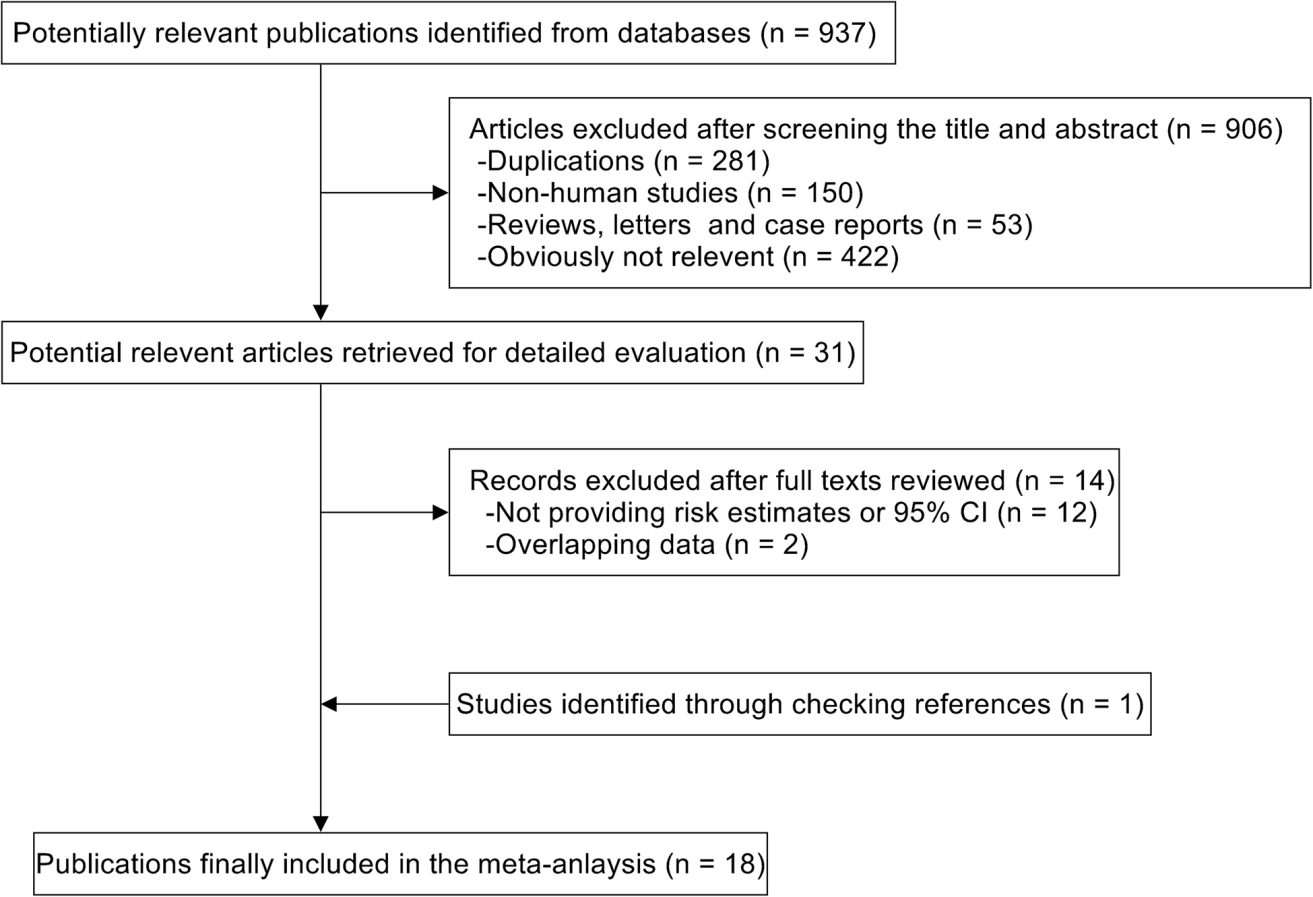
Flowchart of study selection

The detailed steps of literature search were shown in Fig. 1. Briefly, we identified 937 studies through systematic search, of which 906 were excluded after reviewing the titles and abstracts. Thirty-one studies were selected for full-text evaluation, fourteen were further excluded for not providing risk estimates with confidence intervals or overlapping data and 1 study were included by checking references. Finally, eighteen studies were included in this meta-analysis on the association of vitamin B6, B12 and methionine with pancreatic cancer risk [7, 8, 14–17, 24–35]. These studies were published between 1991 and 2020, including eleven prospective studies [7, 8, 15–17, 24–27, 32, 34] and seven case-control studies (comprising a total of 4104 cases) [14, 28–31, 33, 35]. Six studies were performed in US [7, 14, 26, 30, 31, 33], six in Europe [8, 16, 17, 25, 27, 29], five in Asia [15, 28, 32, 34, 35], and one in Australia [24]. Ten studies evaluated association of pancreatic cancer risk with dietary intake of vitamin B6, B12 and methionine [14, 15, 24–27, 29–31, 33], and 8 studies with blood levels of them [7, 16, 17, 25, 28, 32, 34, 35]. The quality scores of the ten studies ranged from 6 to 9. The characteristics of each study included in this meta-analysis were listed in Table 1.

**Table 1:** Observational studies investigating the relationship between vitamin B6, B12, methionine and pancreatic cancer risk

Author (year)	Design	Country	cases	Sex	Source	Exposure	Dose	Adjustment factors	Quality
Baghurst (1991) [24]	PCC	Australia	104	Both	Diet	Vitamin B6 Vitamin B12	Q4 vs Q1 Q4 vs Q1	Age, Sex, energy intake, smoking, alcohol intake	7
Stolzenberg-Solomon (1999) [8]	Nest case-control	Finland	126	Male	Serum	PLP Vitamin B12	> 39.46 nmol/L (T3) vs ≤ 26.34 nmol/L (T1) > 550 pg/ml (T3) vs ≤ 427 pg/ml (T1)	Age, month of blood draw, completion of dietary questionnaire, study center, intervention group, serum folate	7
Stolzenberg-Solomon (2001) [25]	Cohort	Finland	157	Male	Diet	Vitamin B6 Vitamin B12 methionine	> 2.81 mg/d (Q5) vs ≤ 2.09 mg/d (Q1) > 13.68 μg/d (Q5) vs ≤ 7.57 μg/d (Q1) > 2268 mg/d (Q5) vs ≤ 1720 mg/d (Q1)	Age, intervention, folate	7
Skinner (2004) [26]	Cohort	US	326	NHS: women, HPS: men	Diet	Methionine	Q5 vs Q1	Age, time period, and energy,smoking, diabetes, body mass index, and height	6
Schernhammer (2007) [7]	Nest case-control	US	208	NHS: women HPS: men	Plasma	PLP Vitamin B12	Q4 vs Q1 Q4 vs Q1	Age, sex, smoking, fasting status, month of blood draw, physical activity, and a history of diabetes	7
Larsson (2007) [27]	Cohort	Sweden	147	Male	Diet	Vitamin B6 methionine	≥ 2.56 mg/d (Q4) vs < 1.83 mg/d (Q1) ≥ 2.02 g/d (Q4) vs < 1.59 g/d (Q1)	Age, sex, energy intake, smoking, BMI, diabetes	7
Gong (2009) [14]	PCC	US	532	Both	Diet	Vitamin B6 Vitamin B12 methionine	≥ 5.0 mg/d (Q5) vs < 1.9 mg/d (Q1) ≥ 14.5 mg/d (Q5) vs < 4.2 mg/d (Q1) ≥ 2060 mg/d (Q5) vs < 1443 mg/d (Q1)	Age, sex, energy intake, smoking, alcohol intake, diabetes, BMI	7
Guo (2009) [28]	PCC	China	42	Both	Plasma	PLP	High vs low	Age, sex	7
Bravi (2011) [29]	HCC	Italy	326	Both	Diet	Vitamin B6	Q5 vs Q1	Age, sex, and center, and adjusted for year of interview, education, tobacco smoking, history of diabetes, body mass index, and total energy intake	7
Chuang (2011) [16]	Cohort	EU	463	Both	Plasma	PLP Methionine	> 54.82 nmol/L vs ≤ 23.75 nmol/L > 30.24 μmol/L vs ≤ 20.67 μmol/L	Age,sex, education, smoking status, cotinine concentration in plasma, baseline alcohol drinking, BMI and self-reported diabetes status at baseline.	8
Arendt (2013) [17]	Cohort	Denmark	698	Both	Plasma	Vitamin B12	> 800 pmol/L vs 200–600 pmol/L	Not mentioned	5
Jansen (2013) [30]	HCC	US	384	Both	Diet and supplement	Vitamin B6	Q5 vs Q1	Age, sex, energy intake, smoking, alcohol intake, BMI	7
Jansen (2014) [31]	HCC	US	384	Both	Diet and supplement	Vitamin B12 Methionine	Q5 vs Q1 Q5 vs Q1	Age, sex, energy intake, smoking, alcohol intake, BMI	7
Huang (2016) [32]	Cohort	Singapore	271	Both	Diet	Vitamin B6, Vitamin B12 Methionine	1.33 mg/d (Q4) vs 0.88 mg/d (Q1) 3.26 μg/d (Q4) vs 0.88 μg/d (Q1) 1625.25 mg/d (Q4) vs 1073.17 (Q1)	Age, sex, year of interview, dialect group, education, BM, smoking status, diabetes, alcohol drinking, and weekly vitamin use.	9
Huang (2016) [32]	Nest case-control	Singapore and China	187	Both	Serum	PLP	> 52.4 nmol/L vs < 20.0 nmiol/L	Smoking status, alcohol intkaek, level of education, history of diabetes, BMI, and study site	9
Marley (2018) [33]	PCC	US	150	Both	Diet	Vitamin B6, Vitamin B12 Methionine	3.36 mg/d (Q4) vs 1.38 mg/d (Q1) 12.2 μg/d (Q4) vs 2.7 μg/d (Q1) 2.78 g/d (Q4) vs 1.10 g/d (Q1)	Age, sex, race, education, cigarette smoking, alcohol consumption, and total physical activity, energy, total fat, fiber, vegetables, and fruits	8
Nakagawa (2018) [34]	Nested case-control	Japan	170	Both	Plasma	Methionine	34.0 nmol/L (Q4) vs 19.6 nmiol/L (Q1)	Age, sex, PHC area, duration of the fasting period prior to blood sampling, smoking, body mass index, and past history of diabetes mellitus.	8
Huang (2020) [35]	Nest case-control	Singapore and China	187	Both	Serum	Methionine	Q5 vs Q1	Age,sex, level of education, body mass index, smoking status, serum cotinine concentration, number of alcoholic drinkers per week, history of diabetes, serum pyridoxal 5′-phosphate concentration and estimated glomerular filtration rate	9

Abbreviation: *HCC* Hospital-based case-control study, *PCC* Population-based case-control study, *BMI* Body mass index

### Vitamin B6 and PLP levels

**Fig. 2:**
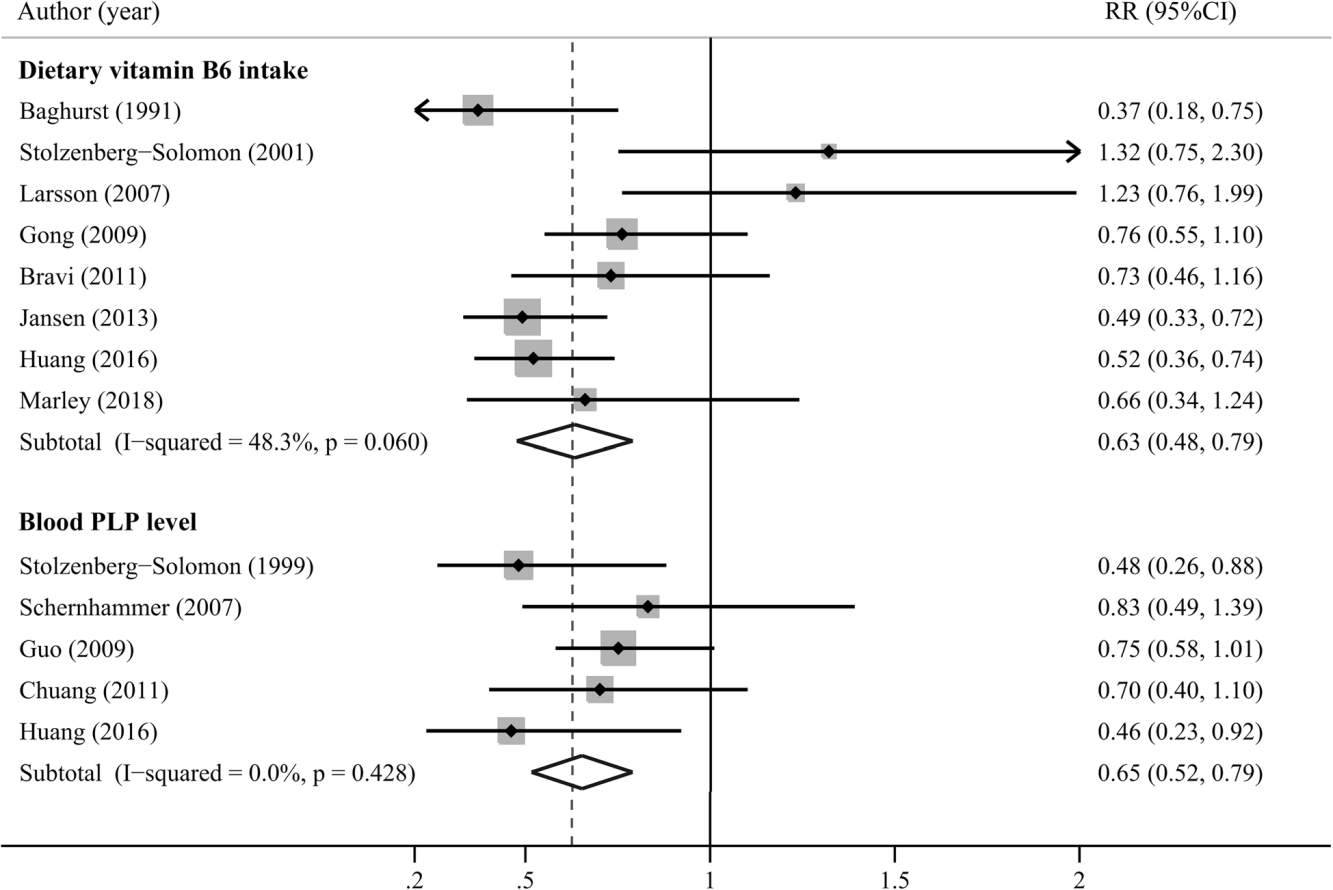
A forest plot of the pooled RR for vitamin B6, blood pyridoxal 5-phosphate (PLP) levels and pancreatic cancer risk

Eight studies reported results on vitamin B6 intake [14, 16, 24, 25, 27, 30, 32, 33], and five studies reported blood PLP levels [7, 8, 16, 28, 32]. The multivariable-adjusted RRs for each study and all studies combined for the highest vs lowest categories of vitamin B6 intake or blood PLP levels are shown in Fig. 2. Results from studies on vitamin B6 intake in relation to pancreatic cancer risk were inconsistent, with moderate heterogeneity (I^2^ = 48.3%, *p* = 0.06). All studies on the association of blood PLP levels with pancreatic cancer risk showed an inverse association, which was statistically significant in 2 studies [8, 32]. No heterogeneity was detected (I^2^ = 0, *p* = 0.428). The pooled RRs of pancreatic cancer for the highest vs lowest categories of vitamin B6 intake and blood PLP level were 0.63 (95% CI, 0.48–0.79) and 0.65 (95% CI, 0.52–0.79), respectively. The Egger’s (*P* = 0.379) or Begg’s (*P* = 0.902, Fig. S1A) test showed no evidence of publication bias for vitamin B6 intake.

We next performed sensitivity analysis to explore the source of the heterogeneity among studies of vitamin B6 intake and pancreatic cancer. The sensitivity analysis removing one study at a time and calculating the pooled RRs for the rest studies showed that no single study substantially influenced the pooled RR (Fig. S2A). Through the Galbraith plot, we noted that 2 studies by Stolzenberg-Solomon and Larsson [25, 27], which reported positive relationships, were the major sources of heterogeneity (Fig. S3A). There was no significant heterogeneity (*P* = 0.361, I^2^ = 8.7%) after excluding the 2 studies, and the inverse association became stronger (OR 0.55; 95% CI, 0.45–0.66). The cumulative meta-analysis is the process of repeated meta-analysis of individual studies each time adding a new study. In the present study, the cumulative meta-analysis sorted by publication year showed no significant association existed before 2011, while a significant association between vitamin B6 intake and risk of pancreatic cancer began to exist and became stable from 2013 (Fig. S4).

In the subgroup analyses, we pooled the RR by study design (cohort or case-control), geographical region (US, Europe, Austrlia and Asia), and number of included cases (≥ 300 or <  300). A statistically significant protective effect of vitamin B6 intake on pancreatic cancer was observed in case-control studies (RR = 0.58; 95% CI, 0.43–0.72), while no such effect in cohort studies (RR = 0.94; 95% CI, 0.35–1.54). Also, the inverse associations were found in US (RR = 0.66; 95% CI, 0.42–0.78), Asia (RR = 0.52; 95% CI, 0.36–0.74) and Australia (RR = 0.37; 95% CI, 0.18–0.75), but not in Europe (RR = 0.99; 95% CI, 0.59–1.38). When stratifying by number of cases, the RR estimates showed vitamin B6 intake was consistently associated with reduced risk of pancreatic cancer (Table 2).

**Table 2:** Subgroup analyses between the intake of vitamin B6, B12 and the risk of pancreatic cancer

	Vitamin B6				Vitamin B12				Methionine			
	N	RR (95%CI)	I^2^(%)	*p-*Value	N	RR (95%CI)	I^2^	*p-*Value	N	RR (95%CI)	I^2^	*p-*Value
Study design												
Cohort	3	0.94 (0.35–1.54)	74.9	0.060	2	0.88 (0.62–1.14)	0	1	4	0.76 (0.49–1.04)	66.2	0.031
Case-control	5	0.58 (0.43–0.72)	24.9	0.256	4	1.05 (0.73–1.38)	36.2	0.195	3	0.90 (0.67–1.12)	0.9	0.365
Geographical region												
USA	3	0.66 (0.42–0.78)	22.2	0.276	3	1.02 (0.65–1.38)	51.1	0.129	4	0.91 (0.73–1.09)	0	0.558
Asia	1	0.52 (0.36–0.74)	–	–	1	0.88 (0.62–1.24)	–	–	1	0.82 (0.57–1.17)	–	–
Europe	3	0.99 (0.59–1.38)	36.1	0.209	1	0.88 (0.53–1.48)	–	–	2	0.67 (0.13–1.20)	71.2	0.062
Australia	1	0.37 (0.18–0.75)	–		1	1.45 (0.74–2.84)	–	–	–	–	–	–
Samples												
≥ 300	3	0.63 (0.44–0.81)	34.3	0.218	4	0.85 (0.63–1.07)	0	0.574	3	0.91 (0.72–1.10)	3.4	0.355
< 300	5	0.68 (0.40–0.96)	61.2	0.035	2	1.19 (0.88–1.50)	0	0.483	4	0.71 (0.42–1.00)	51.5	0.103

**Fig. 3:**
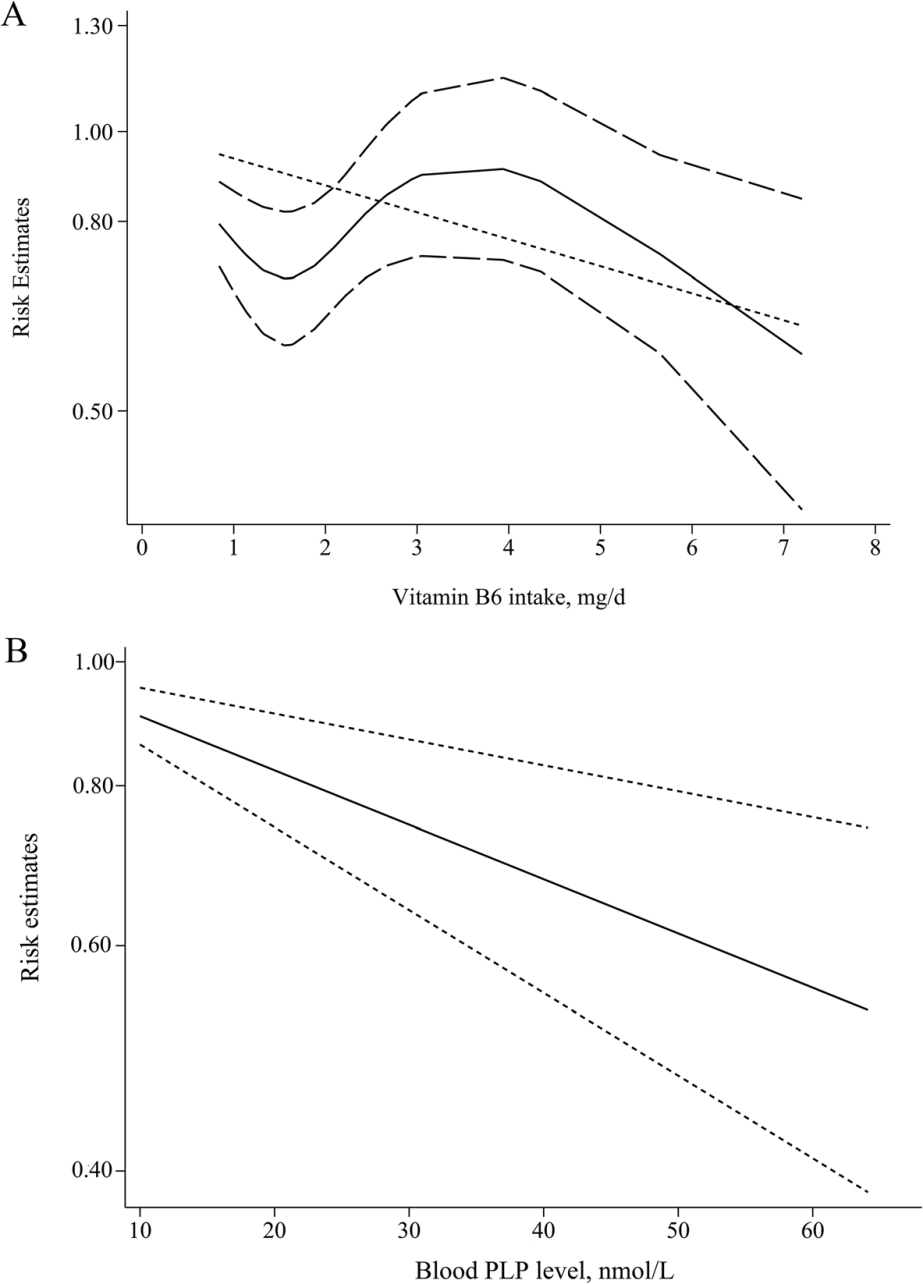
**a** The nonlinear dose-response analysis plot for the vitamin B6 intake and pancreatic cancer risk. The solid line and the long dash line represent the estimated RR and its 95% CI. Short dash line represents the linear relationship. **b** The linear dose-response analysis plot for the blood pyridoxal 5-phosphate (PLP) levels and pancreatic cancer risk. Adjusted relative risks and 95% CIs confidence intervals (dashed lines) are reported. The vertical axis is on a log scale

We evaluated the potential non-linear dose-response relationship between vitamin B6 intake and pancreatic cancer risk. Six studies were included [14, 15, 25, 27, 30, 33] and we found no heterogeneity (P_heterogeneity_ = 0.16) in the overall analysis of vitamin B6 intake, with a significant non-linear dose-response relationship (P_non-linearity_ = 0.002; Fig. 3a). We next assessed the dose-response relationship between blood PLP levels and pancreatic cancer risk. Three studies were analyzed [8, 16, 32] and there was no evidence of statistically significant departure from linearity between blood PLP levels and pancreatic cancer risk (P_non-linearity_ = 0.33). A 10 nmol/ml increment in blood PLP level conferred an RR of 0.91 (95% CI, 0.86–0.95), indicating that the risk of pancreatic cancer was decreased by 9% for every 10 nmol/L increment in blood PLP levels (Fig. 3b).

### Vitamin B12

**Fig. 4:**
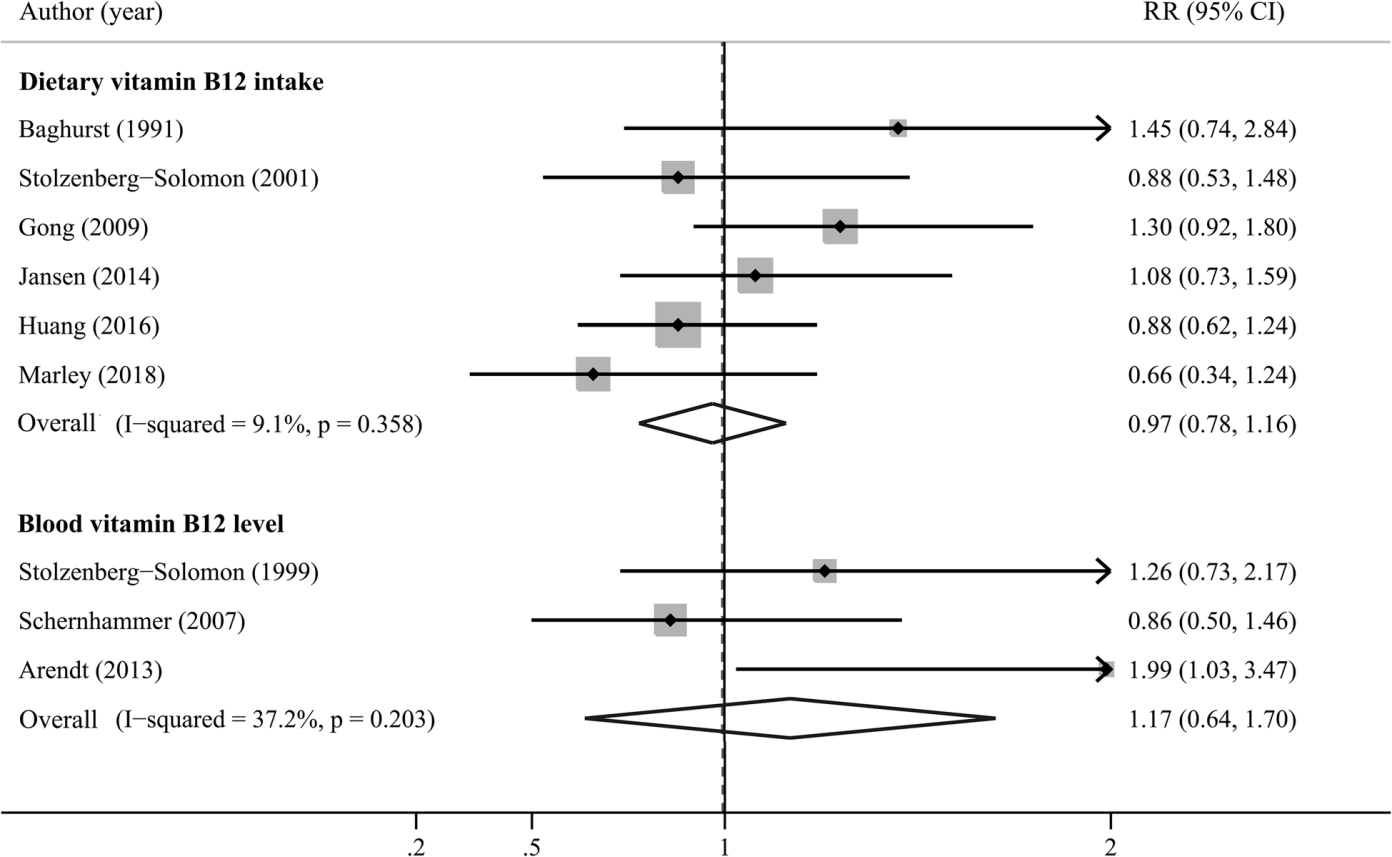
A forest plot of the pooled RR for vitamin B12 intake, blood vitamin B12 levels and pancreatic cancer risk

Six studies reported results on vitamin B12 intake [14, 15, 24, 25, 31, 33], and three studies reported blood vitamin B12 levels [7, 8, 17]. The multivariable adjusted RRs of pancreatic cancer for each study and all studies combined for the highest versus the lowest category of vitamin B12 intake and blood levels are shown in Fig. 4. The summary RRs were 0.97 (95% CI, 0.78–1.16) for vitamin B12 intake and 1.17 (95% CI, 0.64–1.70) for blood levels in a random-effects model, with no evidence of heterogeneity. There was little evidence of publication bias with the association between vitamin B12 intake and risk of pancreatic cancer, as indicated by Begg’s test (*P* = 0.707, Fig. S1B) and Egger’s test (*P* = 0.598). The RR estimates from subgroup analyses varied little, showing no significant association between vitamin B12 intake and pancreatic cancer risk (Table 2).

### Methionine

**Fig. 5:**
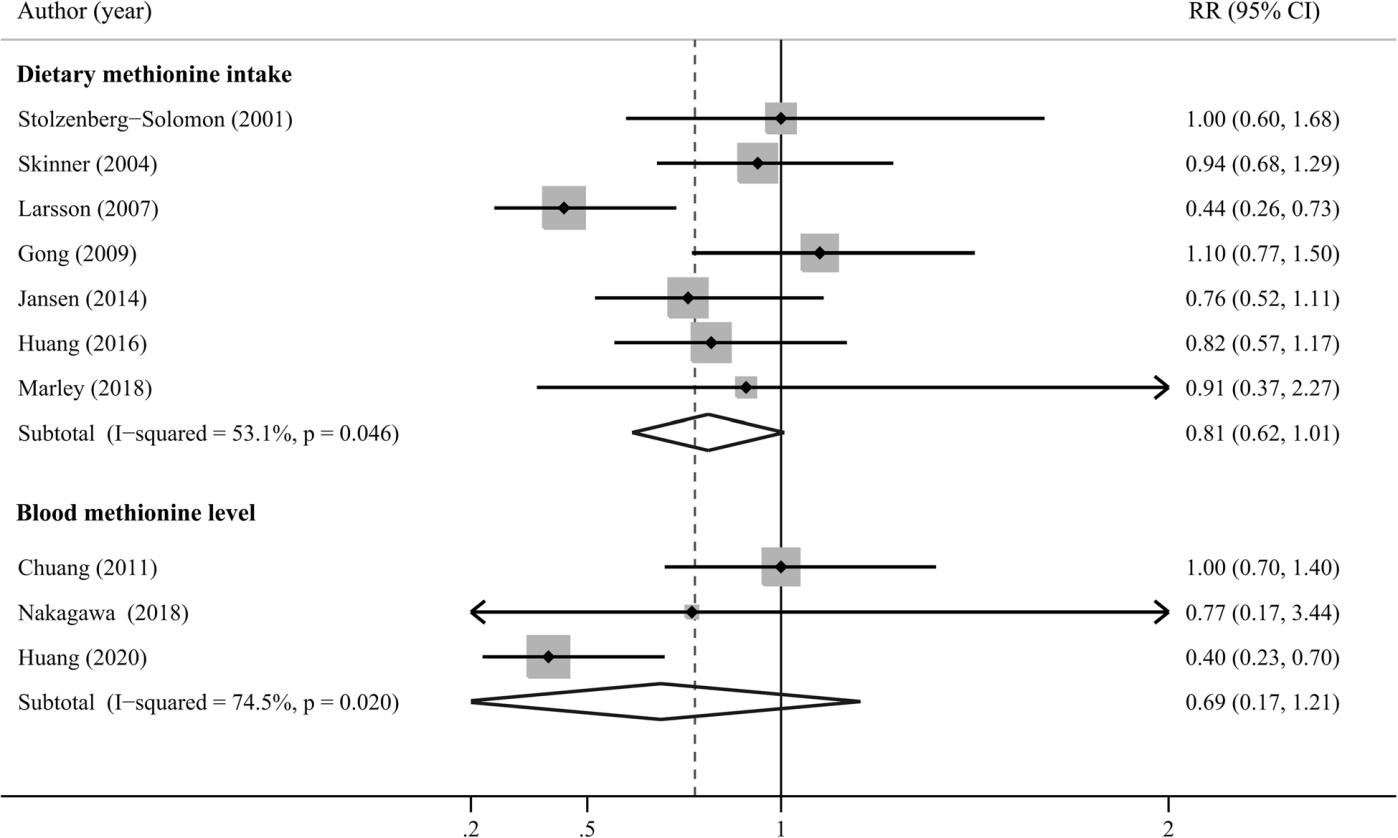
A forest plot of the pooled RR for methionine intake, blood methionine levels and pancreatic cancer risk

Figure 5 presents the results of methionine intake and blood methionine levels and pancreatic cancer risk. Seven studies reported results on methionine intake [14, 15, 25–27, 31, 33], and the summary RR was 0.81 (95% CI: 0.62–1.01) in a random-effects model, with evidence of strong heterogeneity (P_heterogeneity_ = 0.046, I^2^ = 53.1%). The sensitivity analysis indicated that the study by Larsson et al. [27] substantially influenced the pooled OR (Fig. S2B). Also, the Galbraith plot showed that it was the major sources of heterogeneity (Fig. S3B). The summary RR was 0.90 (95% CI, 0.74–1.04), and no significant heterogeneity existed (*P* = 0.779, I^2^ = 0%) after excluding this study. No publication bias was detected by Begg’s (*P* = 0.23, Fig. S1C) and Egger’s test (*P* = 0.748). The subgroup analyses were consistent with the overall results, revealing that methionine intake was not associated with incidence of pancreatic cancer (Table 2). Three studies reported results on blood methionine levels [16, 34, 35], and the summary OR was 0.69 (95% CI: 0.17–1.21), with great heterogeneity (*P* = 0.02, I^2^ = 74.5%).

## Discussion

The association between vitamin B6, B12 or methionine and risk of other cancers has been assessed in previous meta-analysis [36–39]. Wu et al. [38] found that serum PLP levels and methionine intake might be inversely associated with breast cancer risk, while the inverse association was not significant with dietary vitamin B6 intake, serum vitamin B12 levels and dietary vitamin B12 intake. Another meta-analysis of prospective studies indicated that blood PLP levels were inversely associated with risk of colorectal cancer, and there was no significant association between vitamin B6 intake and colorectal cancer risk [37]. The present meta-analysis indicated that increased vitamin B6 intake and levels of its circulating biomarker (PLP) might be significantly associated with reduced risk of pancreatic cancer, but this inverse association was not observed for vitamin B12 and methionine. To the best of our knowledge, this is the first meta-analysis evaluating the relationship between one-carbon metabolic factors and risk of pancreatic cancer.

As for vitamin B6 intake, our results were consistent with a recent meta-analysis, which suggested that vitamin B6 intake could significantly decrease pancreatic cancer risk (RR = 0.65; 95% CI, 0.53–0.90) [40]. However, in that study, there is an obvious mistake that the ORs for the highest vs lowest categories of vitamin B6 intake and serum pyridoxal 5′-phosphate concentrations were combined together in the overall analysis. The present study also included two studies that were omitted in that meta-analysis [24, 26]. Furthermore, our results indicated a non-linear dose-response relationship between vitamin B6 intake and pancreatic cancer risk, but a linear dose-response relationship with the risk of pancreatic cancer decreased by 9% for every 10 nmol/L increase in blood PLP level. In the subgroup analysis, we found a significant risk reduction from case-control studies, but no association from cohort studies, suggesting that our conclusion depend mainly on the case-control studies. Generally speaking, cohort studies provide stronger evidence regarding an association than case-control studies because they are less prone to recall and selection bias. In addition, the non-linear relationship between vitamin B6 intake and pancreatic risk indicated that confounding factors may influence our results. Therefore, great caution should be taken when interpreting the negative association. Further research focusing on this association are warranted to confirm this association.

From a biological point of view, vitamin B6 may play a protective role in the development of pancreatic cancer. Vitamin B6 is a co-factor involved in DNA synthesis and methylation pathway of one-carbon metabolism [9]. Low vitamin B6 intake resulted in a decrease in methylene-THF (methyl donor) production. Global DNA hypomethylation is associated with genomic instability [41] and oncogenesis [42, 43]. It has been reported that dozens of genes were hypermethylated and hypomethylated in pancreatic tumors and cancer cell lines [44].. The decreased methylene-THF pool may also overload the DNA repair system by increasing the binding of uracil to DNA, leading to chromosome breakage [45, 46]. In addition, vitamin B6 can be used as a scavenger of reactive oxidative species. In vitamin B6-deficient rats, the activity of pancreatic glutathione reductase decreased, maintaining the level of glutathione in cells [47]. Glutathione is an antioxidant that maintains the redox state of cells and detoxifies carcinogens, and low glutathione may impair the antioxidant defense system [48]. It is possible that vitamin B6 intake tends to be associated with healthy behaviors that may be protective against pancreatic cancer. However, our results showed that a similar inverse association was also found on blood PLP levels and pancreatic cancer risk. PLP accounts for most of vitamin B6 in the circulation, and are usually used as the main indicator to measure the status of vitamin B6 in the whole body [49]. Vitamin B6 intake has been shown to be reasonably strongly correlated with serum (r = 0.46) [50] and plasma (r = 0.42) [51] PLP levels, respectively. In men, PLP was inversely correlated with urinary 8-hydroxydeoxyguanosine, a marker of DNA oxidative damage [33]. Recent studies have shown that PLP deficiency leads to the formation of advanced glycation end products (AGES), which is the major contributor of oxidative stress and subsequent chromosomal aberrations in Hela cells [52].

We did not observe statistically significant associations between vitamin B12 and methionine and the risk of this malignancy. One potential reason vitamin B12 differs from other B vitamins may be because it is derived exclusively from foods of animal origin, and it is simply a marker for consumption of animal protein, which tends to be associated with unhealthy behaviors that may increase the risk of pancreatic cancer. Previous studies have shown that the risk of meat consumption is positively associated with pancreatic cancer risk [53] and diets low in animal protein can reduce the risk of pancreatic cancer [54, 55]. Regarding methionine, although we found a borderline non-significant risk reduction in the overall population (RR = 0.81; 95% CI, 0.62–1.01), no association was observed by in any subgroup according to study design, geographical region, and number of cases. Considering relatively small number of studies included, additional studies are needed in order to clarify whether methionine plays a role in the carcinogenesis of pancreatic cancer.

The strengths of our study include a comprehensive assessment of one-carbon metabolism-related nutrients. There are also some weaknesses in our study. First, the meta-analysis can’t solve the problem of confounding factors inherent in the study. Lack of control over confounding factors may bias the results in either direction, in which risk estimates are exaggerated or underestimated, although individual studies in this meta-analysis have considered a wide range of potential confounding factors except for the study by Arendt et al. [17]. Second, heterogeneity may be introduced due to methodologic differences, including different exposure levels for the extreme categories (highest versus lowest), exposure range, and dietary assessment methods (interview vs self-administered questionnaire). However, subgroup analysis was not conducted because of limited data availability. Third, we only had three studies reporting specific serum PLP concentration and risk of pancreatic cancer, which may undermine the reliability of the dose-response analysis, although we found a statistically significant association with the risk of pancreatic cancer. In Fig. 5, a significantly reduced risk was observed when vitamin B6 intake is beyond about 500 mg/day. However, the range of vitamin B6 intake included in the dose-response analysis is centered at approximately 130 mg/day to 300 mg/day (Table 1) that may weaken the dose-response relationship at higher levels of vitamin B6 intake. Fourth, since no RCTs was included in this meta-analysis, the time effect of one-carbon metabolism-related nutrients intake on the risk of pancreatic can’t be accurately evaluated. Fifth, one inherent possibility for meta-analysis is publication bias. Publication bias was not found by Begg’s test or Egger’s test in this meta-analysis, however, given the small number of studies in the stratified analysis, the validity of publication bias testing should be interpreted with caution. Finally, this meta-analysis was not submitted to any systematic review register, which might decrease the credibility of the study, although it was reported in accordance with the PRISMA Statement.

## Conclusion

In summary, this present meta-analysis demonstrated that among one-carbon metabolism-related factors, high vitamin B6 intake was associated with lower risk of pancreatic cancer in a non-linear dose-response pattern, and serum PLP level were associated with a significant linear decreased risk of pancreatic cancer. Considering that vitamin B6 is present in a wide variety of foods such as beef, liver, tuna, and bananas, this research is expected to offer novel avenues for the primary prevention and control of pancreatic cancer. However, this evidence is mainly derived from case–control studies and the data for the high level of dietary vitamin B6 intake were sparse, further research including randomized clinical trials is needed to examine the association of dietary vitamin B6 intake with risk of pancreatic cancer at high doses and explore the recommended treatment period to reduce the risk of pancreatic cancer.

## Supplementary information

**Additional file 1.**

**Additional file 2.**

**Additional file 3.**

**Additional file 4.**

## Data Availability

All data generated or analyzed during the current study are included in this published article and its additional files.

## Abbreviations

BMI: Body mass index
CNKI: Chinese National Knowledge Infrastructure
CI: Confidence interval
FFQ: Food frequency questionnaire
HBC: Hospital-based case-control
NOS: Newcastle–Ottawa Quality Assessment Scale
PCC: Population-based case-control
PLP: Pyridoxal 5′-phosphate
RR: Relative risk
