# Supplementary figures and images for: Vitamin B6, vitamin B12 and methionine and risk of pancreatic cancer: a meta-analysis
Source: Nutr J. 2020 Oct 4;19:111. doi: 10.1186/s12937-020-00628-7 (PMC7534168; doi:10.1186/s12937-020-00628-7)

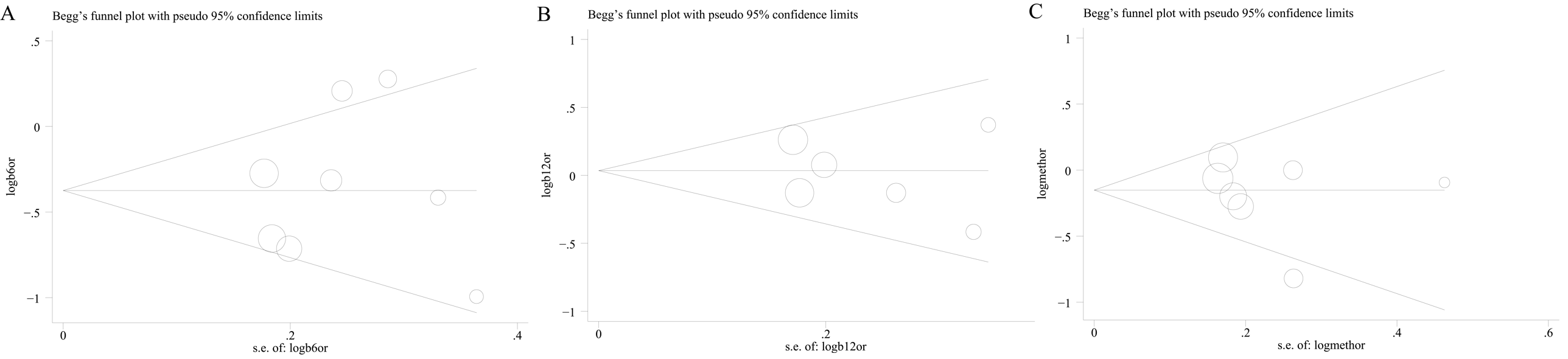

Supplement: Supplementary file 1 — Additional file 1. [file 12937_2020_628_MOESM1_ESM.tif]

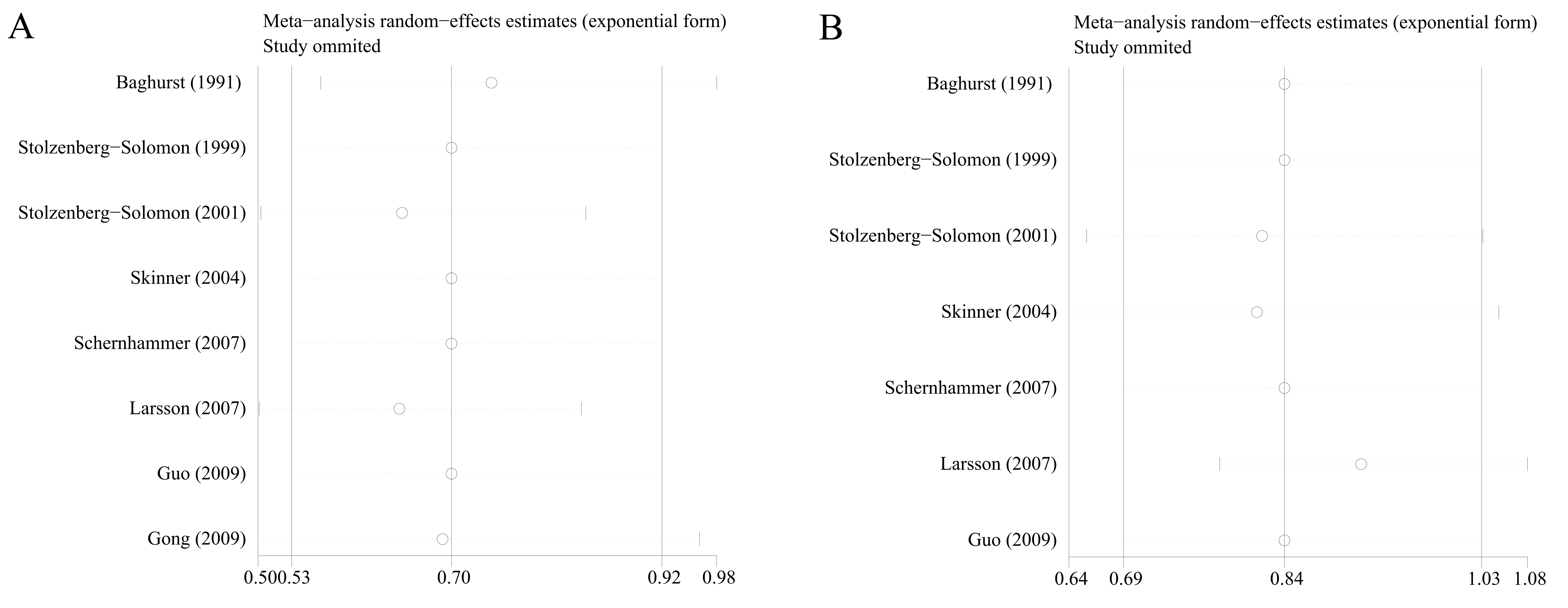

Supplement: Supplementary file 2 — Additional file 2. [file 12937_2020_628_MOESM2_ESM.tif]

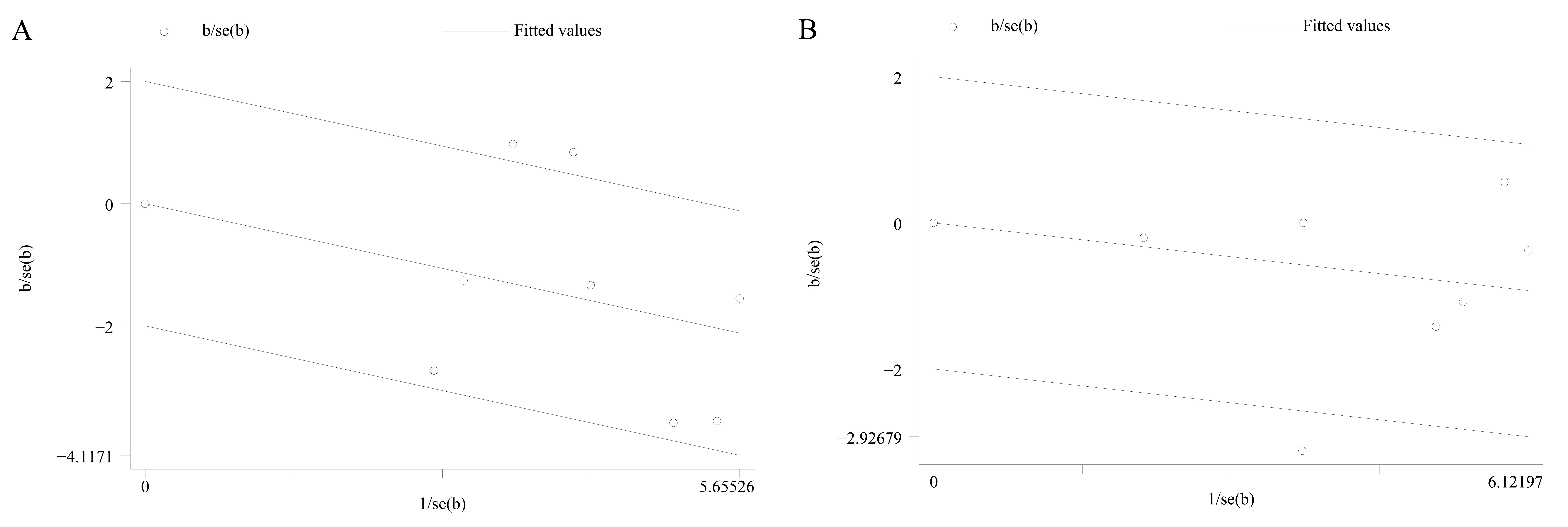

Supplement: Supplementary file 3 — Additional file 3. [file 12937_2020_628_MOESM3_ESM.tif]

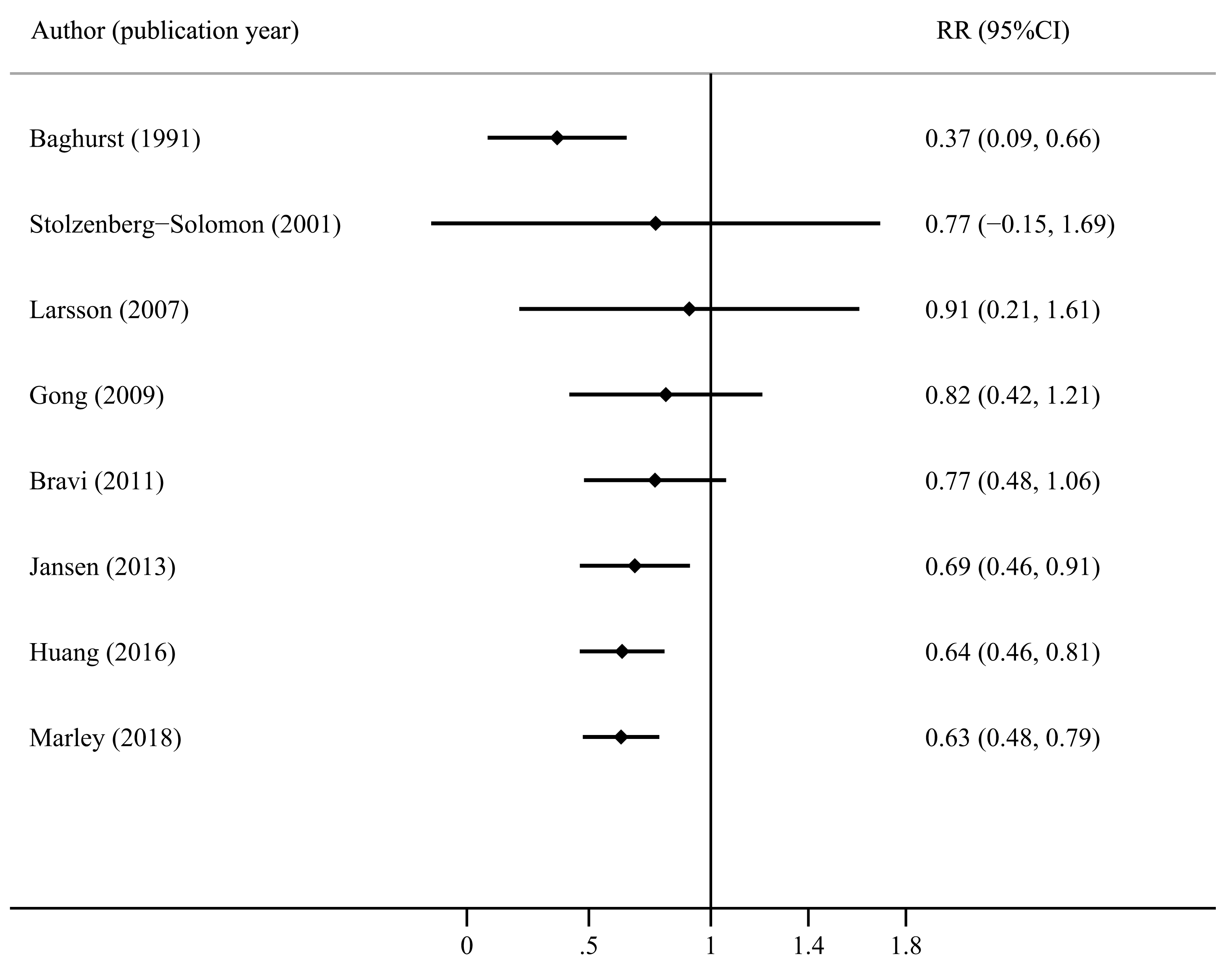

Supplement: Supplementary file 4 — Additional file 4. [file 12937_2020_628_MOESM4_ESM.tif]
